# Gender inequalities across ethnicities in primary care cancer referrals: a scoping review protocol

**DOI:** 10.3399/BJGPO.2023.0211

**Published:** 2024-06-12

**Authors:** Deepthi Lavu, Adnan Khan, Judit Konya, Tanimola Martins, Sarah Price, Richard Neal

**Affiliations:** 1 APEx (Exeter Collaboration for Academic Primary Care), Department of Health and Community Sciences, Faculty of Health and Life Sciences, University of Exeter, Exeter, UK

**Keywords:** primary health care, cancer, gender, sex, inequalities

## Abstract

**Background:**

Early cancer diagnosis is associated with improved mortality and morbidity; however, studies indicate that women and individuals from ethnic minorities experience longer times to diagnosis and worse prognosis compared with their counterparts for various cancers. In countries with a gatekeeper healthcare system, such as the UK, most suspected cancer referrals are initiated in primary care.

**Aim:**

To understand the extent of evidence available on the relationship between primary care cancer referral pathways and cancer outcomes in relation to gender across different ethnic groups. This will identify research gaps and enable development of strategies to ease potential inequalities in cancer diagnosis.

**Design & setting:**

A scoping review of articles written in English, based on the Joanna Briggs Institute methodology. The Preferred Reporting Items for Systematic Reviews and Meta-Analyses extension for Scoping Reviews (PRISMA-ScR) will be used.

**Method:**

Electronic databases and private collections of the team members will be searched for studies. Two independent reviewers will carry out the study selection and data extraction. Based on Population (or participants), Concept, and Context (PCC) framework, this review will consider studies after year 2000, which explored the relationship between gender, across various ethnic groups, and cancer outcomes, following primary care cancer referral in countries with gatekeeper healthcare systems (UK, New Zealand, Sweden, Australia, Canada, Denmark, Republic of Ireland, and Norway). Results will be presented as a narrative analysis.

**Conclusion:**

The results are expected to provide an overview of the discrepancies in primary care cancer referrals based on gender across ethnic groups, which will be crucial to define an appropriate range of strategies to ease any inequalities in primary healthcare cancer diagnosis.

## How this fits in

Health inequalities are unjust and often link to avoidable differences in groups, especially in the context of cancer diagnosis. The results of this scoping review will be used to understand the extent of peer-reviewed literature available on the discrepancies in primary care cancer referrals associated with gender across various ethnic groups. The knowledge that this review may bring is essential to understand the association between gender, across ethnic groups, and cancer outcomes when primary care cancer referral pathways are used. This knowledge will be crucial to identify evidence gaps and possibly an appropriate range of strategies to ease any inequalities in primary healthcare cancer diagnosis.

## Introduction

Cancer has a major impact on society worldwide and results not only in increased morbidity and mortality, but also affects workforce, puts a strain on medical resources, and negatively impacts healthcare costs at individual and national levels.^1^ Early diagnosis and treatment of cancers improve mortality outcomes, and this has understandably led to increased research in the area of early cancer diagnosis.^2,3^ However, this has not translated to advances in avoiding differences in cancer diagnosis across gender and/or ethnic groups. Women have more delays in diagnosis of various cancers compared with men^4–6^and individuals of ethnic minority experienced longer delays than their White counterparts.^6^ Moreover, ethnicity was found to be a significant factor in the stage of diagnosis for women with some cancers.^7^ In addition, some studies reported that women had more emergency presentations with cancer symptoms, which inevitably puts a strain on already overburdened emergency services and is also associated with poorer outcomes.^8^ Despite this literature, evidence regarding the degree of gender discrepancy in cancer referrals and diagnosis is limited.

In the UK, most individuals with cancer symptoms are diagnosed following a referral from primary care, either via the 2-week-wait urgent cancer referral pathway or the elective general practice referral pathway.^9^ Interestingly, although individuals belonging to ethnic minorities were more likely to be referred via the primary care routes for cancer diagnosis,^9^ the time to cancer diagnosis was found to be longer in those groups.^10^ However, gender differences have not been examined in a similar setting, creating the possibility that longer diagnostic intervals for cancers in women were owing to differences in the primary care referral patterns in countries with a gatekeeper healthcare system such as the UK. A study found significant difference in pre-hospital delays in females compared with males for some cancers in the UK but this did not differentiate patient delay from primary care delays.^6^

The objective of this scoping review is to understand the extent of research available and summarise available findings on the relationship between primary care cancer referral pathways and cancer outcomes in relation to gender across different ethnic groups while acknowledging confounders such as age and deprivation. This will help identify gaps in research and enable development of strategies to ease any potential inequalities in primary care cancer diagnosis.

This is the first review, to the best of our knowledge, to examine the specific concept of gender inequalities in cancer diagnosis referral pathways initiated by the primary care team. A preliminary search of MEDLINE, the Cochrane Database of Systematic Reviews, and Joanna Briggs Institute (JBI) Evidence Synthesis was conducted and no current or underway systematic reviews or scoping reviews on the topic were identified. The review question is 'When primary care referral is used as a route to diagnosis of various cancers (such as GP referral pathway and 2-week-wait pathway in the UK or similar routes in other countries), across various ethnic groups:

1. Are time intervals to diagnosis of cancer longer in women than in men?
2. Are emergency presentations more likely in women than in men?
3. Are stage and/or survival for cancers worse in women than in men?'.

## Method

This scoping review will be conducted in accordance with the JBI methodology for scoping reviews.^11^ A research question will be defined, relevant studies identified, studies will be selected, data charted, results will be collated, summarised, and reported, and consultation with stakeholders will be undertaken as part of this review. Preferred Reporting Items for Systematic Reviews and Meta-Analyses (PRISMA) guidelines will be followed, using the PRISMA extension for Scoping Reviews (PRISMA-ScR) checklist.^12^ Literature searching, refining the search strategy, reviewing articles for inclusion, and data extraction will be performed using an iterative approach.

The current protocol follows the JBI guidance.^11^

### Eligibility criteria

The eligibility criteria is elaborated according to the Population (or participants), Concept, and Context (PCC) framework^11^ and the types of sources.

#### Population (or participants)

All manuscripts discussing gender in the primary care cancer referral setting in individuals aged >18 years will be considered in this review.

#### Concept

This review will focus on publications that discuss cancer outcomes of interval of diagnosis and/or emergency presentations and/or stage and/or survival within male and female cohorts (with or without grouping by ethnicity) referred to secondary care by primary care providers in the UK or in other countries with a similar healthcare system. Studies not reporting results by gender or route to diagnosis of cancer will not be included. Studies that focus on other routes of cancer referral, such as screening, emergency, inpatient, outpatient and mixed, and those where the cancer outcomes of interest are not available, will be excluded.

#### Context

Publications within the primary and secondary care health setting discussing the primary care routes of cancer referral will be included in the review. Only studies from the UK, New Zealand, Sweden, Australia, Canada, Denmark, Republic of Ireland, and Norway will be considered as these countries have primary care-led universal access to healthcare. These countries, which formed the International Cancer Benchmarking Partnership, have high-quality and long-standing population-based cancer registration and have broadly comparable wealth and similar healthcare expenditure leading to possible similar policy considerations.^13^

#### Types of sources

All types of quantitative peer-reviewed publications in English will be included if they discuss primary care routes to cancer diagnosis, gender, and cancer outcomes. The review will consider both experimental and quasi-experimental study designs, including randomised controlled trials, non-randomised controlled trials, before and after studies, and interrupted time-series studies. In addition, analytical observational studies, including prospective and retrospective cohort studies, case-control studies, and analytical cross-sectional studies will be considered for inclusion. Descriptive observational study designs, including case series, individual case reports and descriptive cross-sectional studies, will also be considered. Depending on the research question, systematic reviews that meet the inclusion criteria and text and opinion papers will also be considered for inclusion in this review.

### Search strategy

The search strategy of this scoping review consists of recommendations made by the team members and is augmented by strategies suggested by Peters *et al*.^14^ Experienced information specialists from the University of Exeter, UK, were consulted to advise on the search strategy including identification of relevant databases and search terms.

The search strategy will aim to locate published studies. The following electronic databases will be searched: Ovid MEDLINE; CINAHL; Ovid Embase; and CENTRAL. An initial limited search of Ovid Embase and CINAHL was undertaken to identify articles on the topic. Text words contained in the titles and abstracts of relevant articles, and index terms used to describe the articles were used to develop a full-search strategy for Embase. The search string for the Ovid Embase, which will be adapted for other databases as appropriate, is presented in Supplementary Box 1. The database searches will be conducted using controlled vocabulary terms – for example, MESH and free-text terms for the following six concepts: cancer; gender; ethnicity; primary care; study type; and outcomes. These will be combined with the Boolean operators 'AND' or 'OR'.

Private collections of the team members will be searched to include relevant manuscripts. This is a part of our search strategy as our team includes some very experienced researchers in the field of primary care cancer diagnosis. Once we identify the included articles from the database searching, we will forward and backward citation chase using Scopus.

Only peer-reviewed publications published since the year 2000 will be included in this review to keep the results current and also as the 2-week wait system was introduced in England in that year following the Department of Health’s NHS Cancer Plan (2000). Manuscripts in languages other than English will be excluded to avoid translation-associated errors.

### Study and source of evidence selection

Following the search, all identified citations will be uploaded into EndNote (version 20/2013(Clarivate Analytics, PA, USA)) for removal of duplicates before transferring to Covidence software (Veritas Health Innovation, Melbourne, Australia) for title and abstract screening. Following a pilot test, titles and abstracts will be screened by two or more independent reviewers for assessment against the inclusion criteria. Potentially relevant sources will be retrieved in full and will be assessed in detail against the inclusion criteria by two or more independent reviewers. Reasons for exclusion of any full-text articles will be recorded and reported. Any disagreements regarding study eligibility in both the above steps will be resolved either by consensus or by consultation with an additional reviewer. The results of the search and the study inclusion process will be reported in full and presented in a PRISMA-ScR flow diagram.^12^

### Data extraction

Data extraction from the final selected papers will be carried out by two or more independent reviewers using a modified JBI data extraction tool developed by the reviewers to allow gathering data specific to the objectives of the review. The data extracted will include specific details about the PCC study methods and key findings relevant to the review question. A draft extraction form is provided (Supplementary Box 2). Planned piloting of the draft extraction form will be carried out at the beginning of the data extraction process and modifications will be made to the tool as necessary during the data extraction. Modifications will be detailed in the review. Any disagreements between the reviewers will be resolved through discussion, or with an additional reviewer(s). If appropriate, authors of papers will be contacted to request missing or additional data, where required.

### Methodological quality assessment

No risk of bias assessment will be undertaken as such assessments are not typical of scoping reviews.

### Data analysis and presentation

A PRISMA flowchart will summarise our search strategy. Data extracted will be presented in a tabular form using headings similar to how they were extracted and the information will be synthesised to understand the extent of studies on gender discrepancies across various ethnic groups if present. The data extraction items will be analysed by quantifying the text and frequency counts of the data items. A narrative summary of the data extracted will describe how the results relate to the review question. A discussion will include review of our objectives considering the results found. Research gaps will be identified and their implications for future work and policy explored. Dissemination of research outputs will be undertaken in the form of a peer-reviewed publication, presentation of lay abstracts with images in conferences and meetings, and engaging social media content for the public.

### Consultation and collaboration

Our team consists of people with varied experience and interests including primary care doctors, researchers with interest in ethnic inequalities, epidemiologists, and those with special interest in women’s health. We worked in partnership with public collaborators from the Peninsula Public Engagement Group (PenPEG) and public collaborators, which are a part of the Exeter Collaboration for Academic Primary Care (APEx) patient and public involvement group, to develop the study question and the review protocol. We will continue to work with public collaborators throughout the study period with a view to making the research relevant to the population. The aim is that this wide range of involvement will bring varied views to our literature analysis.

## Discussion

With this scoping review, we aim to map knowledge about associations between gender and cancer outcomes, across both ethnic groups and primary care cancer referral pathways. This will help us identify and analyse knowledge gaps in the literature on this topic, including areas where fewer data are available, allowing for identification of future research initiatives to bridge these gaps. Another anticipated implication of this study for practice and research is that synthesising this evidence may allow the identification of appropriate strategies and interventions to address gender and ethnic inequalities in early cancer diagnosis arising through use of the suspected cancer referral pathway. Identifying gender inequalities in cancer diagnostic pathways could impact the timely identification and management of cancer in individuals at risk, making care safer and improving cancer prognosis, which will have an undeniable impact on population health. We hope that this synthesised knowledge can bring important contributions to practice and research by developing new lines of research and influencing professionals and decision makers.

## Trial registration number

This scoping review protocol is registered in Open Science Framework: https://osf.io/jvtxb. Registration DOI: https://doi.org/10.17605/OSF.IO/JVTXB
